# Supplementary material for: Self-DNA Early Exposure in Cultivated and Weedy Setaria Triggers ROS Degradation Signaling Pathways and Root Growth Inhibition
Source: Plants (Basel). 2023 Mar 13;12(6):1288. doi: 10.3390/plants12061288 (PMC10058795; doi:10.3390/plants12061288)

Supplementary Materials

Supplementary Table S1. Primers used in real-time qPCR analysis. For each source gene, the Phytozome ID, strand and position on the genome are shown, with the forward and reverse primer sequences and either the reference paper for the primer, or the reference tool used to design it.

Source Gene	Phytozome ID and position	Primer (5′-3′)	Reference/Tool
FSD2	Seita.4G031200<br>plus strand<br>Scaffold_4:2020702..2023953	Fwd: TGGTTGGGTTTGGCTTGTCTTG<br>Rev: TGTCCCAAGAGATGAGATGGTCCA	(Primer3web v.4.1.0)
ALDH22A1	Seita.2G440100<br>minus strand<br>Scaffold_2:48874364..48880144	Fwd: CAAGAAGAGGCATTTGGACC<br>Rev: TTGATTGCTGCTACACCACAG	(Zhu *et al*., 2014 [49])
ALDH7B1	Seita.2G218400<br>minus strand<br>Scaffold_2:32046039..32052337	Fwd: TCTGCGGAAACTGTGTTGTC<br>Rev: TGAACCATTAGACCAGCCCT	(Zhu *et al*., 2014 [49])
CSD3	Seita.9G403600<br>minus strand<br>Scaffold_9:46291516..46295065	Fwd: CTCAAGCCTGGCCTCCACGG<br>Rev: CAGTGGGATCTGGCTGTCGGT	(Primer3web v.4.1.0)
WD40-144	Seita.6G076200<br>minus strand<br>Scaffold_6:6733603..6739573	Fwd: TACCATCTCGCACGCTACAGGTTT<br>Rev: TCCATGCAACCATCATCACCGACT	(Primer3web v.4.1.0)
WD40-155	Seita.6G247500<br>minus strand<br>Scaffold_6:35501203..35506269	Fwd: TCAAGGAGGAGAACGAGGTGCAC<br>Rev: GCAGCGCCATAACCCTCACCA	(Primer3web v.4.1.0)
MPK17	Seita.4G273900<br>plus strand<br>Scaffold_4:39101196..39106532	Fwd: CGAGAGCCACAGGAAGAACTCAGT<br>Rev: CCTGTGCGGGTATCTACTGCTGC	(Primer3web v.4.1.0)
RNA Polymerase II	Seita.2G142700<br>plus strand<br>Scaffold_2:17011362..17018255	Fwd: TAGGAAAGGAATTGGCAAGG<br>Rev: TAGGACTGCTTTCGACCCA	(Kumar *et al*., 2013 [55])

Supplementary Table S2. Results of the cross-factorial experiment. Data refer to the inhibition tests on root elongation of the two *Setaria* species exposed to purified extracellular DNA solutions at different concentrations and from different sources. For each treatment combination (target species, DNA source and DNA concentration) mean and standard deviation of root elongation (expressed as percentage of the control) are shown. Different small letters in brackets indicate significant differences among combinations of target species and DNA source within each concentration level (i.e. table column, see also lettering in Figure 1 in main text), whereas different capital letters in brackets indicate significant differences among combinations of DNA source and concentration within each target species (table blocks). All pairwise comparisons were tested with Tukey's post hoc test (α = 0.05). Asterisks indicate means significantly different from the control (one-sample t test with Bonferroni's correction for multiple comparisons). The symbol § indicates borderline p-values (0.002 < P < 0.05) in the treatment vs. control tests.

**Target Species**	**DNA source**	**DNA Concentration (ng/µl)**		
		**2**	**10**	**50**
S. italica	*S. italica*	58.47 ± 3.20 (a, CD) *	27.44 ± 2.53 (a, AB) *	14.91 ± 3.06 (a, A) *
	S. pumila	93.18 ± 8.93 (b, F)	72.68 ± 6.38 (bc, DE) §	46.06 ± 4.23 (b, BC) *
	B. napus	89.68 ± 7.45 (b, EF)	68.81 ± 2.67 (bc, D) §	67.91 ± 7.69 (c, D) §
	S. salar	104.48 ± 7.88 (b, F)	98.45 ± 2.84 (d, F)	95.81 ± 7.88 (d, F)
S. pumila	*S. italica*	104.98 ± 7.20 (b, F)	87.99 ± 5.00 (cd, EF) §	50.26 ± 4.29 (bc, BC) *
	S. pumila	70.08 ± 6.88 (a, DE) §	39.63 ± 6.89 (a, AB) *	24.64 ± 3.95 (a, A) *
	B. napus	105.68 ± 6.41 (b, F)	68.32 ± 6.17 (bc, CD) §	63.88 ± 9.46 (bc, CD) §
	S. salar	104.42 ± 5.75 (b, F)	97.57 ± 3.12 (d, F)	92.15 ± 6.41 (d, F)

Supplementary Table S3. Results of the real-time qPCR test on *S. italica* and *S. pumila* exposed to self-DNA. Data refer to mean and standard deviation of ΔCq calculated over 3 biological replicates for each species, target gene and exposure time. Mean and standard deviation of ΔCq observed in the unexposed controls are also shown. Different letters indicate significant differences between 1 h and 3 h ΔCq for each species and gene, as resulting from two-independent-sample t test with Bonferroni's correction for multiple comparisons. Asterisks indicate means significantly different from control (two-independent-sample t test with Bonferroni's correction). The symbol § indicates borderline P-values ($0.00238 < P < 0.05$) in the treatment vs. control comparisons.

**Species**	**Target Gene**	**1h**	**3h**	**Control**
S. italica	SiFSD2	1.259 ± 0.169 *	1.380 ± 0.133 *	2.464 ± 0.103
	ALDH22A1	-0.938 ± 0.148 *	-0.778 ± 0.104 *	-0.131 ± 0.156
	ALDH7B1	-1.855 ± 0.197	-1.740 ± 0.119	-1.685 ± 0.194
	CSD3	-3.807 ± 0.203	-3.885 ± 0.156	-3.507 ± 0.193
	WD40-144	-0.867 ± 0.073	-0.523 ± 0.073 §	-0.954 ± 0.127
	WD40-155	1.495 ± 0.068 *	1.795 ± 0.102 *	-0.562 ± 0.116
	MPK17-1	-0.374 ± 0.127 (a) §	0.990 ± 0.094 (b) *	0.018 ± 0.156
S. pumila	SiFSD2	1.782 ± 0.191 *	1.495 ± 0.123 *	3.060 ± 0.194
	ALDH22A1	-1.533 ± 0.169 *	-1.189 ± 0.170 §	-0.631 ± 0.088
	ALDH7B1	-1.112 ± 0.182	-1.053 ± 0.156	-0.931 ± 0.165
	CSD3	-4.851 ± 0.027 (a) *	-5.332 ± 0.106 (b) *	-4.427 ± 0.115
	WD40-144	-0.810 ± 0.127	-0.769 ± 0.130 §	-1.040 ± 0.131
	WD40-155	1.575 ± 0.084 (a) *	2.165 ± 0.085 (b) *	0.069 ± 0.173
	MPK17-1	-0.063 ± 0.150 (a) *	1.474 ± 0.184 (b) *	0.639 ± 0.168

Supplementary Figure S1. Interaction plot for the term S × D of the ANOVA model presented in Table 1 in main text.

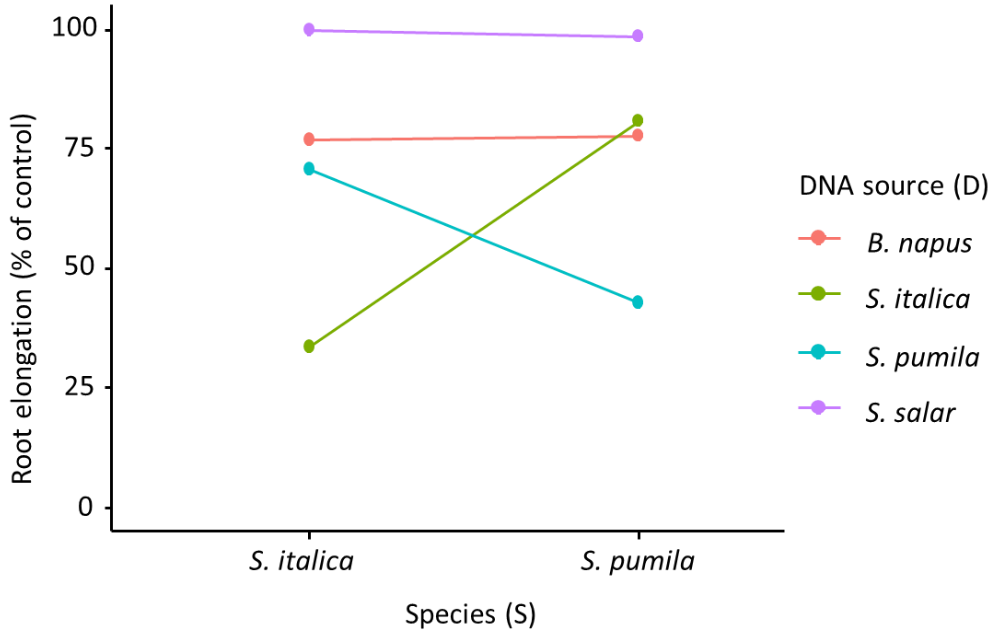

Supplementary Figure S2. Interaction plot for the term D × C of the ANOVA model presented in Table 1 in main text.

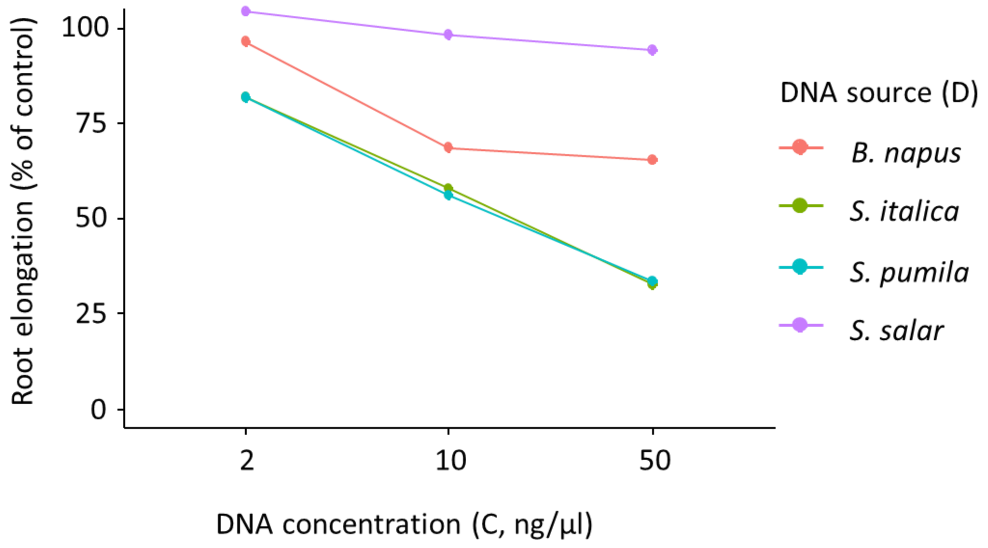

Supplement: Supplementary file 1 [file plants-12-01288-s001.zip › plants-2150959-supplementary.pdf]
